# Characterization of Nme5-Like Gene/Protein from the Red Alga *Chondrus Crispus*

**DOI:** 10.3390/md18010013

**Published:** 2019-12-21

**Authors:** Dragutin Perina, Marina Korolija, Andreja Mikoč, Mirna Halasz, Maja Herak Bosnar, Helena Ćetković

**Affiliations:** 1 Division of Molecular Biology, Ruđer Bošković Institute, 10000 Zagreb, Croatia; mikoc@irb.hr (A.M.);; 2 Ministry of Health of the Republic of Croatia, 10000 Zagreb, Croatia; 3 Division of Molecular Medicine, Ruđer Bošković Institute, 10000 Zagreb, Croatia; mkorolija@mup.hr (M.K.); mherak@irb.hr (M.H.B.); 4 Forensic Science Centre “Ivan Vučetić”, 10000 Zagreb, Croatia

**Keywords:** Nme gene/protein family, Nme5, red alga, *Chondrus crispus*, eukaryotic evolution, Nme evolution

## Abstract

The Nme gene/protein family of nucleoside diphosphate kinases (NDPK) was originally named after its member Nm23-H1/Nme1, the first identified metastasis suppressor. Human Nme proteins are divided in two groups. They all possess nucleoside diphosphate kinase domain (NDK). Group I (Nme1-Nme4) display a single type NDK domain, whereas Group II (Nme5-Nme9) display a single or several different NDK domains, associated or not associated with extra-domains. Data strongly suggest that, unlike Group I, none of the members of Group II display measurable NDPK activity, although some of them autophosphorylate. The multimeric form is required for the NDPK activity. Group I proteins are known to multimerize, while there are no data on the multimerization of Group II proteins. The Group II ancestral type protein was shown to be conserved in several species from three eukaryotic supergroups. Here, we analysed the Nme protein from an early branching eukaryotic lineage, the red alga *Chondrus crispus*. We show that the ancestral type protein, unlike its human homologue, was fully functional multimeric NDPK with high affinity to various types of DNA and dispersed localization throughout the eukaryotic cell. Its overexpression inhibits both cell proliferation and the anchorage-independent growth of cells in soft agar but fails to deregulate cell apoptosis. We conclude that the ancestral gene has changed during eukaryotic evolution, possibly in correlation with the protein function.

## 1. Introduction

The Nme/Nm23/NDPK is a gene/protein family of nucleoside diphosphate kinases (NDPK), enzymes that transfer the terminal phosphate group from (d)NTP (mainly ATP) to any (d)NDP to produce (d)NTPs. In the late eighties it was discovered that one of the family members, Nm23-H1/Nme1/NDPK A, suppresses metastasis formation and was, therefore, identified as the first metastasis suppressor gene/protein [1]. To date, ten different *Nme* genes have been identified in humans. *Nme1* and *Nme2* are the most studied representatives due to their involvement in cancer progression and metastasis. Nme proteins have been divided into two groups based on their phylogenetic characteristics, presence of protein domains and exon/intron structure [2]. Group I and Group II constitute human Nme1-Nme4 and Nme5-Nme9 proteins, respectively. All Nme enzymes possess at least one nucleoside diphosphate kinase (NDK) domain. There is a single histidine residue involved in the catalytic mechanism, conserved in all known active NDPK enzymes. Active NDK domain acts via a ping-pong mechanism, in which the phosphorylation of the histidine residue occurs by transfer of the terminal phosphate group from (d)NTP. These phosphoenzymes can than transfer the phosphate group to any (d)NDP to produce (d)NTP. Group I proteins display a single type NDK domain, whereas Group II proteins display a single or several NDK domains of different types, sometimes accompanied by extra-domains. The Nme10 protein has passed through a separate evolutionary history since it seems evident that its NDK domain was inserted relatively recently and, therefore, cannot be classified in either of the two groups [2]. Interestingly, it seems that the basic function of NDPKs to form NTPs and dNTPs is only inherent to Group I members [3]. To date none of the tested Group II Nme family members were able to transfer terminal phosphate group from (d)NTP to (d)NDP, with a potential exception of Nme6 [4]. At least one of the representatives of Group I proteins is present in all three domains of life. In eukaryotes, members of the Group I proteins are enzymatically active in their hexameric form. The Group I proteins show different cellular localization. Nme1/Nme2 are localized in the cytosol and in the nucleus, Nme4 is exclusively mitochondrial, while Nme3 is considered to be both cytoplasmic and mitochondrial [5]. Interestingly, the Nme genes/proteins have several additional biochemical roles. It has been suggested that they may function as transcription regulators [6], protein kinases [7], and DNases [8]. They also possess diverse biological roles in the cell, including proliferation [9,10], development [11,12], differentiation [13,14], ciliary functions [15], endocytosis and membrane remodeling [16,17], actin-based cellular remodeling [18], apoptosis [8], cell–cell communication [19], and cell adhesion [20,21]. In spite of extensive scientific research in this field for more than two decades, the mechanisms by which the members of the Nme protein family execute their biological functions are still not elucidated.

In contrast to Group I members, the accumulating data suggest that none of the Group II members display measurable NDPK activities [3,22,23,24], even though some of them are able to autophosphorylate [3,23]. The multimeric forms of Group II members have not yet been determined. Some features like the three amino acid insertion in the *Kpn*-loop (named after the “Killer of prune” mutation of the Drosophila Awd NDP kinase) present in all human Group II members, indicate the lack of stability of the multimeric structure potentially resulting in defective catalytic activity [24]. Although little is known about the Group II members’ biology it seems that they might have highly divergent functions. Nme5 has been found to be most prominently expressed in human testes. It localizes within axonemal microtubules of spermatid and spermatozoan flagella, suggesting its involvement in sperm motility [24,25]. Nme5 was found to be strictly associated with active spermatogenesis in both mouse and lizard [26]. Additionally, expression of *Nme5* in mice increased the cellular levels of GPX-5 (glutathione peroxidase-5), which suggests its critical role in eliminating reactive oxygen species during spermatogenesis [27].

Comprehensive proteomic analysis of isolated human ciliary axonemes revealed that Nme5 is also present in somatic airway epithelia cells, together with Nme7 and Nme9 [28]. Furthermore, Nme5 has been identified as a contributor of innate resistance to gemcitabine [29,30]. It has, also, been shown that simvastatin treatment of human prostate cancer cell line (PC3) resulted in reduced protein levels of Nme5 [31]. Expression analyses of 23 purine metabolism genes in Alzheimer’s showed deregulation at the mRNA level of 10 genes, including *Nme1, Nme3*, *Nme5*, *Nme7*, and adenylate kinase 5 (*AK5* [32]).

Vertebrate Nme5 possesses a C-terminal Dpy-30 domain of generally unknown function. In *Chlamydomonas reinhardtii,* this domain and its flanking sequence mediate the assembly and modulation of flagellar radial spoke complexes [33]. In *Caenorhabditis elegans* the DPY-30 protein is essential for dosage compensation in early embryogenesis, while in males (X0) it is required for developmental processes such as coordinated movement, normal body size, correct tail morphology and mating behavior [34]. In mammals, this Dpy-30 is involved in the histone H3K4 methylation and cell fate specification of embryonic stem cells [35].

The human Nme6 was reported independently by two groups in 1999 [36]. Compared to Nme1 and Nme2, Nme6 has additional residues on the N and C termini and an insertion in the *Kpn*-loop. The fusion protein with a GST tag showed some kinase activity, although lower compared to human Nme1 or Nme2 [4]. Our results on Nme6-like protein in sponges suggest that the function of *Nme6* gene has probably changed during metazoan evolution in correlation with the structure of the protein [22]. Recent analyses of melanoma driver genes revealed that *Nme6* has a significant enrichment of somatic mutations rate [37]. Further, it has been shown that both Nme6 and Nme7 are important in embryonic stem cell renewal [38].

The most intriguing result considering Group II members came up upon the discovery that recombinant human Nme7 induces a stable, naïve-like state in human embryonic stem cells and induces pluripotent stem cells [39]. Nme7^−/−^ mice developed situs inversus and hydrocephalus, which indicates a role of Nme7 in biogenesis or a function in motile cilia [40]. Normal male fertility and the absence of rhinitis/sinusitis observed in Nme7^−/−^ mice indicates the importance of Nme7 in biogenesis of ependymal or embryonic node cilia, rather than the role in respiratory epithelium and spermatozoa cilia/flagella [40]. In vitro co-sedimentation assays using recombinant proteins showed that both mouse and *Chlamydomonas* Nme7 directly bind to microtubules [41], suggesting its ancient function that might be universal for all eukaryotes. Human Nme7 contains a DM10 domain of unknown function followed by two NDK domains of which only the first one is able to autophosphorylate [23]. An identical fluorescence pattern of both DM10 alone and the full length Nme7 (TcNDPK2) GFP-fused products were detected linked to the cytoskeleton and flagellum of *Trypanosoma cruzi,* which gives the first insight into the role of the DM10 domains in Nme enzymes [42].

Human Nme8 is an ortholog of the sea urchin IC1 that encodes a component of sperm outer dynein arms, and is implicated in primary ciliary dyskinesia, left–right asymmetry randomization, and male infertility [43].

*Nme9* gene displays a similar exon/intron structure as *Nme8*, and therefore it seems likely that *Nme9* originates from an incompletely translocated duplication of *Nme8* [2]. Both proteins possess thioredoxin domain at the N-terminus. Nme8 is distributed in human sperm [44], where it is probably required during the final stages of sperm tail maturation [45]. On the other hand, Nme9 is a microtubule-binding protein expressed predominantly in the cilia of lung airway epithelium and spermatid manchette and axoneme [46]. Recently, the association between *Nme8* locus polymorphism and Alzheimer’s disease has been demonstrated [47,48].

Our comprehensive analyses of the Nme family in eukaryotes revealed conservancy of the Group II ancestral type proteins in several species from three eukaryotic supergroups. We analyzed Nme proteins from early branching eukaryotic lineage. The red algae (Rhodophyta) are one of the oldest groups of eukaryotic algae formed during the primary endosymbiosis event which enabled the emergence of the first photosynthetic eukaryote [49]. The red macroalgal fossil, 1.2 billion years old, provides the oldest evidence of a multicellular, sexually reproducing eukaryote [50]. The genome of *Chondrus crispus*, Irish moss, has been sequenced, which was a prerequisite for the positioning of red algae as excellent model organisms for understanding Nme evolution [51]. Our analyses revealed that the ancestral type of Nme5-like protein, in contrast to the human homologue, was a fully functional multimeric NDP kinase. This three-domain protein shows high affinity to various types of DNA and displays a dispersed localization throughout the eukaryotic cell. Simple bioassays revealed that cells overexpressing this enzyme exhibit inhibition of proliferation and anchorage independent growth in soft agar but show no influence on apoptosis. We concluded that the structure of the ancestral gene has changed significantly during eukaryotic evolution in correlation with the protein function.

## 2. Results

### 2.1. Nme Family in Eukaryotes

The increasing number of sequenced eukaryotic genomes reveals that the Nme superfamily distribution is wider than previously documented. We analyzed the distribution of Nme proteins in all six major eukaryotic supergroups: Opisthokonta, Amoebozoa, Plantae, Excavata, Chromalveolata, and Rhizaria. We found Nme homologues from both Group I and Group II in the representatives of all major eukaryotic supergroups (Table 1). Broader analyses of Nme enzymes, revealed that 1448 Nme proteins are present in 398 eukaryotic species across all six eukaryotic supergroups. In all these species, 37 types of Nme enzymes were found. The majority of these proteins possess the same combination of domains present in the human Nme homologues, but there are also many additional distinct domains, suggesting novel connections between the Nme family and different cellular processes.

**Figure 1:**
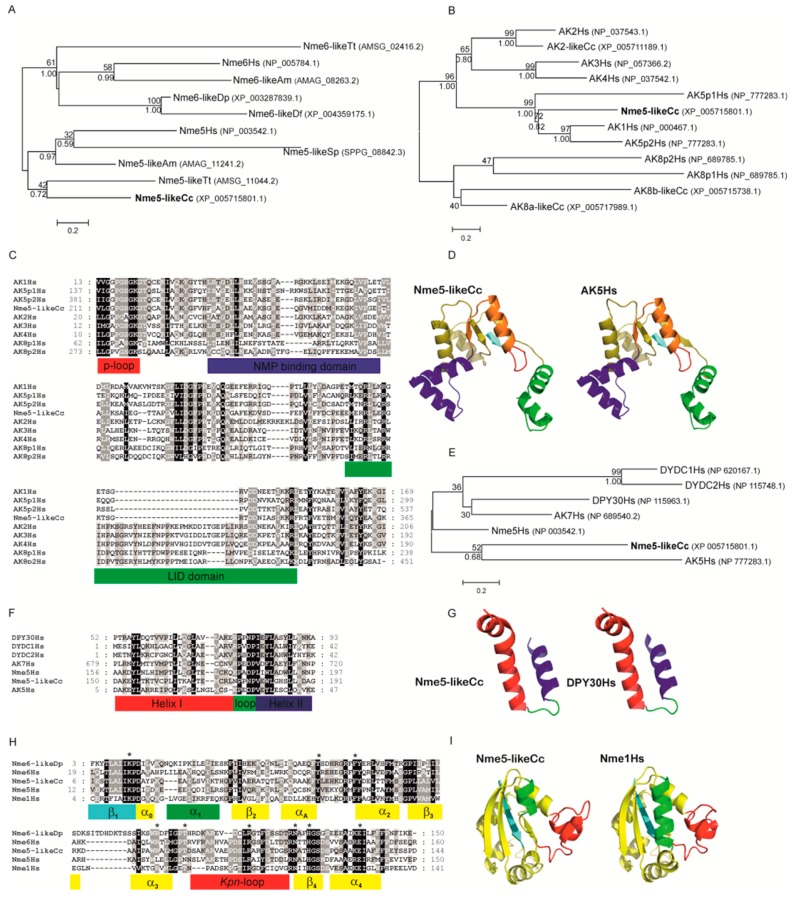
Maximum likelihood phylogenetic trees illustrating relationship between NDK (**A**), ADK (**B**) and Dpy-30 (**E**) domains from Nme5-likeCc and protein domains from selected representatives. MCMC (Markov chain Monte Carlo) values are given below lines, bootstrap values above and accession numbers are given after protein names and species abbreviation. The scale bar indicates genetic distance of the branch lengths. Amino acid sequence alignments of ADK (**C**), Dpy-30 (**F**), and NDK (**H**) domains from representative species are accompanied with colored bars which show major motifs in these domains. Nine amino acid residues important for quaternary structure, nucleotide binding and catalysis are marked with asterisk. Homology modeling of the ADK (**D**), Dpy-30 (**G**), and NDK (**I**) sequences from Nme5-likeCc against the crystallographic algorithms of the human AK5 (PDBID: 2BWJ), DPY30 (PDBID: 3G36) and Nme1 (PDBID: 3L7U), respectively. Shortened species names include Hs, *Homo sapiens*; Cc, *Chondrus crispus*; Tt, *Thecamonas trahens*; Dp, *Dictyostelium purpureum*; Am, *Allomyces macrogynus*; Df, *Dictyostelium fasciculatum*; Sp, *Spizellomyces punctatus*.

In representatives from all eukaryotic supergroups analyzed, more than one Group I *Nme* gene was found. It was previously proposed that duplications of Group I *Nme* genes occurred independently and frequently in metazoan evolution. However, the evolutionary relations were generally not well resolved [2,53,54,55]. As in our earlier studies, phylogenetic analyses of Group I members (data not shown) do not produce a well-supported tree, and therefore we can only speculate on evolutionary relations between duplicated *Nme* Group I genes present in distant eukaryotic supergroups. Recent human *Nme1-4* genes group together, and are generated by duplication events that occurred in the vertebrate lineage [2,53]. On the contrary, Group II members seem to have emerged earlier in the eukaryotic evolution. It has been proposed that *Nme5*, *Nme6*, and *Nme7* were most likely present in the eukaryote ancestor [55]. Our phylogenetic analyses, however, show that *Nme6* probably arose in the Unikonts lineage (Figure 1A). *Nme6*-like gene is present in Apusozoa representative who is presumably the closest relative of Opisthokonts, and in Amoeboza representatives (Table 1). *Nme6*-like gene seemingly arose by the duplication of *Nme5*-like in the ancestor of Unikonts, probably before the separation of Amoebozoa (Figure 1A). It was proposed that *-Nme8* is a choanoflagellate/metazoan innovation [55]. We found *Nme8*-like genes also in Fungi (Table 1). *Nme8*-like appeared earlier in Unikont evolution than previously suggested, probably in the ancestor of all Opisthokonts, after the separation of Apusozoa. *Nme5*-like and *Nme7*-like seem to be widely distributed throughout eukaryotic supergroups which indicates that the ancestor of all eukaryotes probably had both genes. The only exception is the secondary loss within Amoebozoa lineage, a supergroup without any sequenced representative, containing neither *Nme5*-like nor *Nme7*-like. In the majority of eukaryotes analyzed Nme7-like protein possesses the same domain structure as its human homologue, which indicates that the eukaryotic ancestor probably possessed *Nme7*-like gene with an identical domain organization structure. On the contrary, the distribution of Nme5-like revealed that the eukaryotic ancestor probably possessed a Nme5-like domain organization dissimilar to the human homologue. Basal representatives from three eukaryotic supergroups also possess an adenylate kinase (ADK) domain present at the C-terminal end of Nme5-like (Table 1). Adenylate kinases are phosphotransferase enzymes present in all three domains of life and they are a part of the cellular machinery responsible for nucleotide synthesis. So far, nine different adenylate kinase isoenzymes have been identified and characterized in human. Nme5-like from red algae *C. crispus* (Nme5-likeCc) possesses an ADK domain that groups together with human AK1/AK5 (Figure 1B). It is comprised of well conserved elements essential for the phosphotransferase activity: the glycine rich region (P-loop), (d)NMP binding site, and LID domain, which is responsible for the substrate ((d)NTP or (d)NDP) binding (Figure 1C).

Homology modeling of the ADK sequence from Nme5-likeCc against the crystallographic algorithms of the human AK5 (Protein Data Bank ID 2BWJ) demonstrated similar monomeric structures (Figure 1D). Human AK5 possesses Dpy-30 domain at the N-terminus, which may indicate that recent human AK1/AK5 clade arose when ancestral three-domain Nme5-like split into Nme5 and AK5. Phylogenetic analyses of the Dpy-30 domain support this thesis as Nme5-likeCc groups with human AK5, rather than with any other human Dpy-30 protein (Figure 1E). Dpy-30 domain seems to be well conserved, with hydrophobic residues and prolines present in all analyzed enzymes from human and red algae (Figure 1F). The modeled Dpy-30 domain from the Nme5-likeCc is comprised of a helix-loop-helix structure identical to the human Dpy-30 domain (Figure 1G). The two amino acid deletion observed in the human Nme5 and the Nme5-likeCc sequence located in the α1 helix and three amino acid insertion in the *Kpn*-loop (Figure 1H) cause significant structural changes in monomer NDK structure, compared to Nme1 which may prevent hexamer assembly and/or enzymatic activity (Figure 1I). However, the ATP binding cavity in the hexameric structure of Nme5-likeCc seems to be much more conserved than it is the case in human Nme5 (Supplementary File Figure S1). Although, NDPK domains are not significantly conserved (Supplementary File Table S1), Nme5-likeCc possesses the NDK domain with all nine residues essential for ATP-binding, quaternary structure and catalysis, unlike the inactive human Nme5 homologue. Therefore, we produced recombinant Nme5-likeCc to test its activity.

### 2.2. The Ancestral Nme5-LikeCc Protein Forms a Multimer

**Figure 2:**
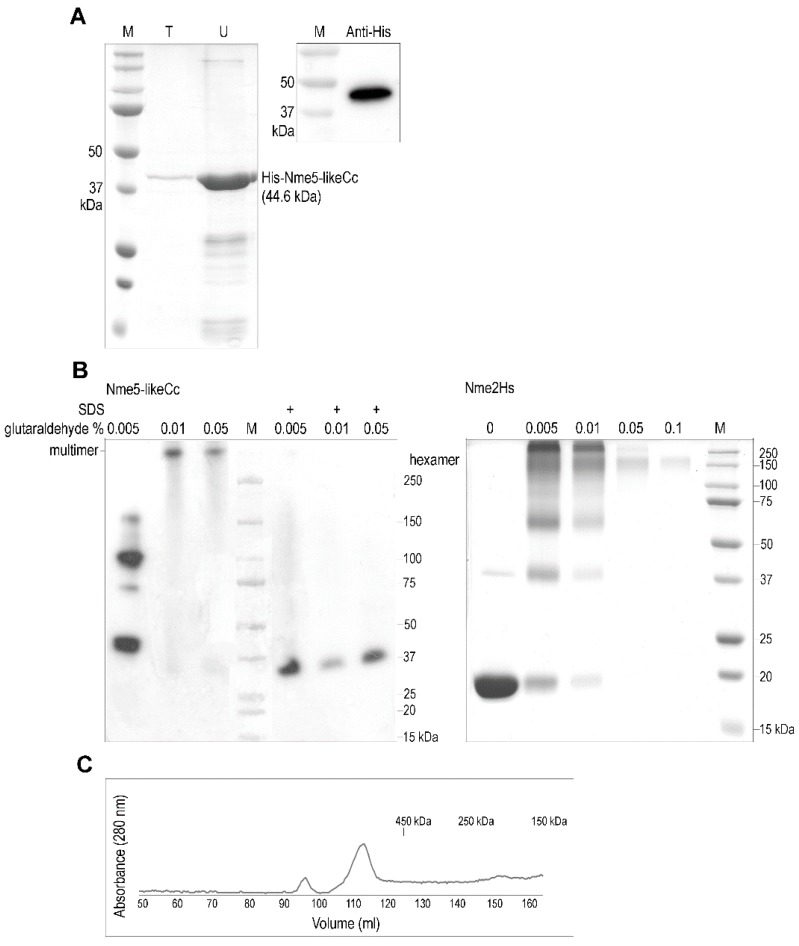
Purification and oligomerization of Nme5-likeCc protein. (**A**) Recombinant His-Nme5-likeCc, purified with cobalt-charged agarose column—TALON (T), concentrated and desalted using Ultracel (U) was blotted against anti-His antibody (1:5000) and visualized using chemiluminescence detection. (**B**) Cross-linking of Nme5-likeCc protein. Purified recombinant protein Nme5-likeCc (300 ng) was pre-incubated in PBS at room temperature with glutaraldehyde to initiate the cross-linking. After quenching, the reaction product was subjected to 12% SDS-PAGE, transfer on PVDF Hybond-P membrane, incubated with anti-His antibody (1:5000) and visualized using chemiluminescence detection. Human Nme2 protein (Nme2Hs) was used as control. (**C**) Size exclusion chromatography. Recombinant Nme5-likeCc was loaded onto HiLoad 26/600 Superdex 200 prep grade column and eluted with Nme buffer at a flow rate of 1 mL/min. The standard protein markers for the gel filtration chromatography were as follows: ferritin (440 kDa), β-amylase (200 kD) and alcohol dehydrogenase (150 kD).

Approximately 10 µg of soluble recombinant Nme5-likeCc protein was obtained from 1 L of bacterial culture (Figure 2A). Most of the protein was insoluble and ended up in the pellet fraction. The multimeric structure is required for the NDPK activity [14,56,57]. Eukaryotic Nme enzymes that belong to Group I form trimers of parallel dimers forming an active hexamer. Dissociated subunits of these Nme enzymes do not possess NDPK activity, although every monomer possesses one catalytic site [14]. Multimeric structure of any Group II Nme member is not yet determined or not even predicted, although it is speculated that the trimeric association might be greatly altered in human Nme5, and it is strongly in favor of a dimeric structure [24]. A high-molecular (>250 kDa) oligomeric structure was found after cross-linking the Nme5-likeCc protein with glutaraldehyde. Previously described human Nme1 was used as control (Figure 2B). The multimeric structure of Nme5-likeCc was also confirmed by gel filtration (Figure 2C).

### 2.3. Recombinant Nme5-LikeCc Possesses NDPK Activity and Binds Nonspecifically to Various Types of DNA

The kinase activity of the recombinant Nme5-likeCc was quantified using a PK-LDH coupled method that detects (d)ADP/(d)GDP as product of the phosphotransfer reaction between (d)ATP/(d)GTP and dTDP nucleotides. The specific kinase activities of the Nme5-likeCc enzyme were measured and found to be 273 ± 36 U/mg, 184 ± 14 U/mg and 225 ± 24 U/mg, when ATP, dATP and GTP were used as phosphate donors, respectively. In comparison, the NDP kinase activity of Nme proteins ranged from 208 U/mg to 2700 U/mg [58]. The specific activities of the Nme5-likeCc kinase were similar and within the previously reported range for the human Nme1/2 protein. This protein is the first Group II Nme family member with similar NDPK activity as the Group I Nme proteins. Interestingly, purified recombinant Nme5-likeCc is devoid of detectable NDP kinase activity when dGTP was used as the phosphate donor.

**Figure 3:**
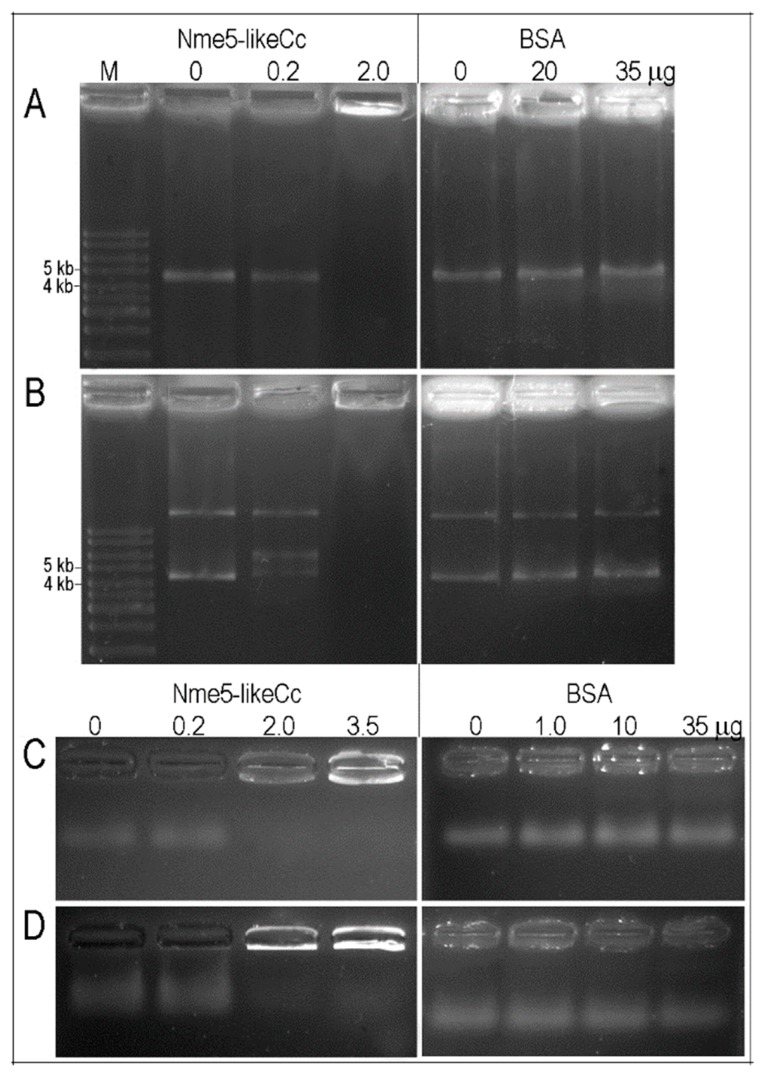
DNA-binding activity of the Nme5-likeCc protein. The reaction was performed in buffer containing 100 mM KCl, 10 mM MgCl_2_, 50 mM Tris HCl pH 7.5, 0.2 mM DTT, 10 µg/mL BSA and (**A**) 10 ng of single-stranded circular DNA or **(B**) 10 ng of double-stranded circular DNA or (**C**) 30 ng of single-stranded 36-bp DNA or (**D**) 30 ng of double-stranded 57-bp DNA and purified Nme5-likeCc protein as indicated. BSA was used as control. Products were analyzed in 1% agarose gel (**A**,**B**) or 4% agarose gel (**C**,**D**) and stained with SYBR gold. Notice that protein-DNA complex remain trapped in the wells at 2 and 3.5 µg protein amounts.

Nme proteins are able to bind nonspecifically and sequence-specific single-stranded linear/circular DNA and/or double stranded DNA. For example, Nme2 is involved in DNA structural changes necessary for the activity of the *c-myc* promoter [59]. To test binding activities, Nme5-likeCc protein was incubated with various types of DNA; single-stranded circular DNA from the bacteriophage φX174, double-stranded circular DNA (bacteriophage φX174 RF I), double-stranded 57-bp nuclease hypersensitive element of the *c-myc* promoter and single-stranded telomeric DNA (TTAGGG)_6_. The recombinant protein displayed the ability to bind to all tested DNA types, showing the DNA band retardation effect (Figure 3).

### 2.4. The Ancestral Nme5-LikeCc Protein Localizes in Nucleus and Cytoplasm

**Figure 4:**
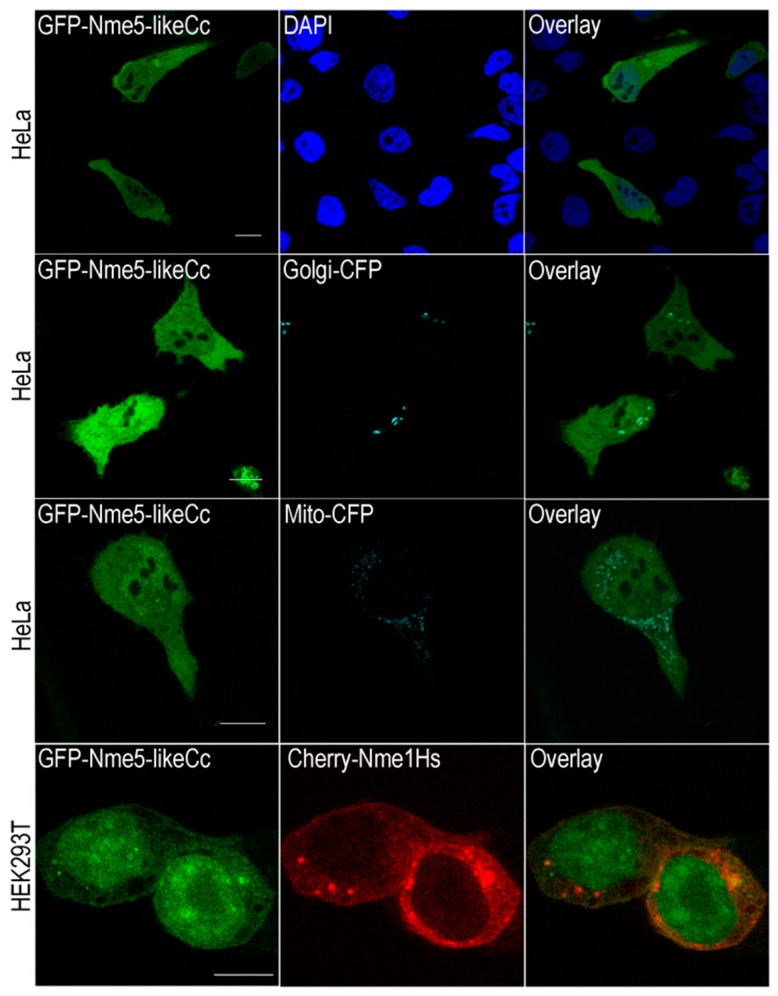
Subcellular localization of Nme5-likeCc. HeLa and HEK293T cells were transiently transfected with pEGFP-Nme5-likeCc (green fluorescence), pCherry-Nme1Hs (red fluorescence), pECFP-Golgi (cyan) and pECFP-mitochondria (cyan). The overlay (yellow) indicates colocalization of the human Nme1 and Nme5-likeCc. Scale bar = 10 μm.

Nme enzymes are mainly cytoplasmic, but can be found, at least transiently, associated with membranous structures and in the nuclei [15]. Several of them (Nme3, Nme4 and Nme6) localize in mitochondria, at least partly. Nme4 possesses the canonical mitochondrial signaling sequence and exclusively localizes in this organelle [52,60]. Human Nme7 was recently identified as a centrosomal protein [23], while Nme5 was found to be microtubule associated [25]. We transiently transfected HeLa and HEK293T cells with GFP-Nme5-likeCc and analyzed the cells 48 h post transfection using confocal laser scanning microscopy (Figure 4). Since ancestral Nme5-likeCc seems to have characteristics of an active Nme enzyme, unlike the recent human homologue Nme5, we investigated its localization in comparison to other active NME members, namely Nme1. Human Nme1 is mainly localized in the cytoplasm and to a lesser extent in the nucleus of HEK293T, which is consistent with a previous study [61]. Protein GFP-Nme5-likeCc is dispersed throughout the cytoplasm rather than associated with a specific organelle. The overlay (yellow signal) indicates colocalization of the human Nme1 and Nme5-likeCc in the cytoplasm. A more pronounced nuclear localization for the GFP-Nme5-likeCc in HEK293T cells can be seen very clearly (Figure 4).

### 2.5. The Ancestral Nme5-LikeCc Protein Diminished Colony Growth in HEK293T Cell Line and Inhibited Cell Proliferation

**Figure 5:**
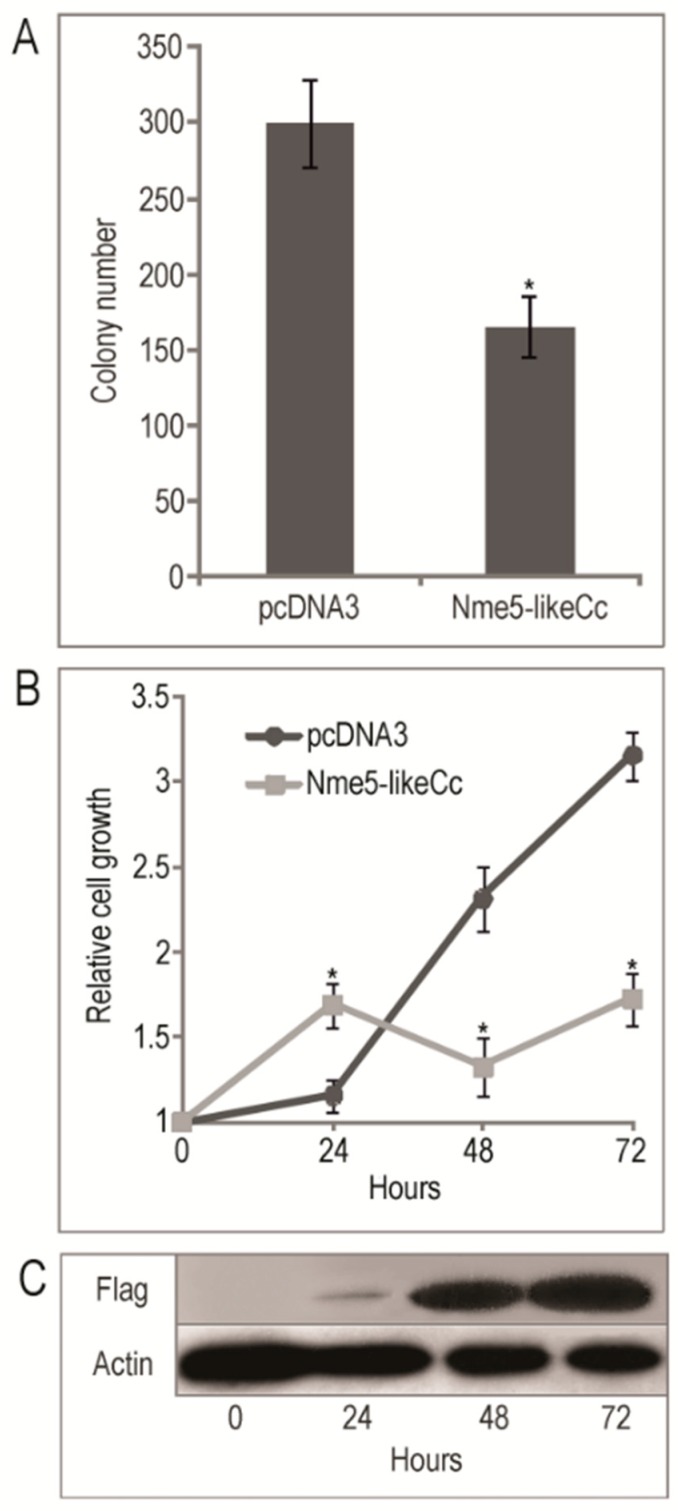
(**A**) Quantitative results of soft agar colonization assay with HEK293T cells transfected with pcDNA3-Nme5-likeCc or empty vector pcDNA3 (**B**) The relative growth rates assessed by CellTiter-Glo assay of HEK293T cells transfected with pcDNA3-Nme5-likeCc or empty vector pcDNA3 and protein blot validation of Flag-Nme5-likeCc expression (**C**). Data are representative of three independent experiments (* *p* < 0.05).

It has been demonstrated that the human Nme1 suppresses anchorage independent growth in soft agar in human breast adenocarcinoma cell lines MDA-MB-435 and MDA-MB-231. Moreover, Nme2, Nme4 and Nme5 significantly diminished colony growth in both cell lines [61]. Another study showed that stably-transfected chronic myeloid leukemia K562 cells, expressing RNAi for post-transcriptional silencing of *Nme1* gene, exhibited a markedly increased ability to form colonies in terms of number and size compared to the control [62]. Furthermore, it has been established that the murine neuroblastoma cell line N1E-115 expressing Nme3 formed significantly fewer colonies in soft agar [63], while both Nme2-expressing NIH3T3 fibroblasts and Nme2-expressing HLK3 hepatocytes form colonies, unlike control cells that do not form any [64]. Compared to control, stable transfectants of human neuroblastoma NB69 cell lines expressing Nme1 and mutant Nme1S120G form 2.8 and 3.6 times more colonies on soft agar, respectively, indicating that Nme1 aberrations provide neuroblastoma cells with additional in vitro colonization ability [65]. Since Nme family members seem to have an impact on soft agar colony forming in various cell types, we tested Nme5-likeCc prospective suppression. In comparison to control cells, the transfected HEK293T cells exhibited decreased ability to form colonies on soft agar by 1.8-fold (*p* = 2.69 × 10^−6^) (Figure 5A). The colonies formed by the Nme5-likeCc HEK293T cells were significantly smaller, (the largest were composed of 25 cells) compared to the colonies formed by the control empty vector (mostly >40 cells). Colonies with more than 20 cells were counted. Further, we analyzed the effect of Nme5-likeCc on cell proliferation. The growth rate of cells was evaluated at different time points after transfection, to determine whether the overexpression of Nme5-likeCc affects cell proliferation (Figure 5B). The pcDNA3 ”empty” vector was used as control. The transfection markedly increased Flag-Nme5-likeCc levels (Figure 5C). Subsequently, the proliferation of pcDNA3-Nme5-likeCc-transfected HEK293T cells decreased 1.83-fold (*p* = 0.0004) comparing to cells with pcDNA3 control (Figure 5B). The cell viability decreased by 45.4% 72 h post-transfection compared to cells possessing the control vector. The results presented in Figure 5B demonstrate that while the Nme5-likeCc inhibits proliferation of HEK293T cells tested at 48 h and 72 h post-treatment, it enhances cell proliferation 1.46-fold (*p* = 0.005) when compared to cells with pcDNA3 control 24 h post-transfection. The results of these assays show that the Nme5-likeCc modulates cell proliferation in the HEK293T cell line.

## 3. Discussion

**Figure 6:**
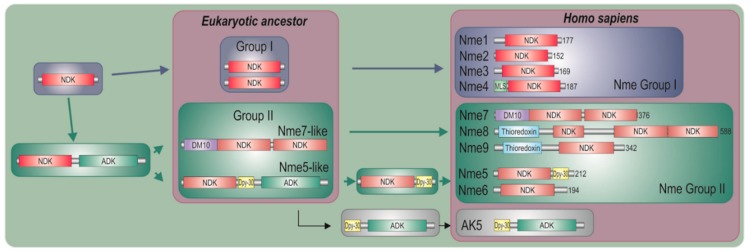
Proposed schematic model for the evolution of recent human Nme Group II enzymes.

Distribution analyses indicate that the ancestor of all eukaryotes probably possessed two Group II members of Nme family of proteins, namely Nme5-like and Nme7-like. The domain organization of Nme5-like protein from red algae *C. crispus* probably reflects the structure of the ancestral protein (Figure 6). Phylogenetic analyses revealed that the C-terminal end of Nme5-likeCc is most similar to recent metazoan AK5 protein. We propose a hypothesis that the ancestral gene split in two genes very early in the eukaryotic evolution but remained conserved in several basal eukaryotic representatives. The human AK5 is cytosolic, or both nuclear and cytosolic [66] expressed almost exclusively in the brain, the pons and the spinal cord. This active kinase phosphorylates AMP, dAMP, CMP, and dCMP using ATP or GTP as phosphate donors [67]. Interestingly, it can also use ATP and GTP to phosphorylate CDP, GDP, UDP, dADP, dCDP, dGDP, and TDP and, like many other adenylate kinases, acts like an NDP kinase [68]. The adenylate kinase complements NDPK function in the *ndk*-disrupted strains of *Escherichia coli* [69]. The NDP kinase is absent in *Ureaplasma parvum* genome, but *U. parvum* possesses five nucleoside monophosphate kinases (NMPKs): adenylate kinase (ADK), cytidylate kinase (CMPK), uridylate kinase (UMPK), guanylate kinase (GMPK), and thymidylate kinase (TMPK). ADK, CMPK, UMPK, and TMPK from *Ureaplasma* were purified and characterized and it was discovered that these NMPKs are base specific and are capable of converting (d)NMPs directly to (d)NTPs [70]. These intriguing connections between ADK and NDPK enzymes can be further elucidated by the analyses of the ancestral Nme5-likeCc protein. Future site-directed mutational studies should explain whether its NDPK activity is due to the active ADK domain and if the NDK domain lost its NDPK activity very early in the evolution of eukaryotes. The crystal structure of this multimeric enzyme should reveal interactions between NDK and ADK domains that are essential for its activity and confirm if these interactions still exist, even after the ancestral gene split (e.g., interactions between human Nme5 and AK5). Our analyses showed that Nme5-likeCc protein can bind to various types of DNA. It can bind to single-stranded telomeric DNA (TTAGGG)_6_, to the double-stranded 57-bp nuclease hypersensitive element of the *c-myc* promoter, and also to the single-stranded circular DNA from bacteriophage ϕX174 like human Nme2 [53,59,71]. It is yet to be determined whether this affinity to various types of DNA is sequence specific. Nonspecific DNA binding by Nme5-likeCc is not incompatible with potential sequence-specific DNA-binding because, for example, human transcription factor Nme2 also binds the DNA sequence nonspecifically, particularly when the substrate DNA is single-stranded [72]. It should also be elucidated if the more pronounced nuclear localization of the EGFP-Nme5-likeCc in HEK293T cells is associated with its potential transcriptional or some other nuclear activity.

Hexameric conformation is required for stability as well as for efficient enzyme catalysis. The site-directed mutagenesis and X-ray structures of Nme enzymes identified nine amino acid residues crucial for quaternary structure, nucleotide binding and catalysis [24]. All of these residues are conserved in Nme5-likeCc (Lys-14, Tyr-52, Phe-60, Arg-88, Thr-94, Arg-108, Asn-118, His-121 and Glu-132). In human inactive Nme5, Leu-94 and Asn-100 replace Arg and Thr residues, respectively which are involved in the interaction with the β-phosphate and the maintenance of the quaternary structure [24]. It was also shown that Ser-120 and Pro-96 in Nme1 protein are important for hexameric oligomerization [73]. The serine (Ser-123) residue is conserved in Nme5-likeCc. The insertion of three amino acids in the *Kpn*-loop present in the human Nme5 is evident in the ancestral Nme5-likeCc, as well. This insertion is located near Pro-96 (Nme1). Thus, we can conclude that although all the residues necessary for the hexameric structure are present in Nme5-likeCc enzyme, and known residues that could impede this structure are absent, the insertion of three amino acids seems to have an impact on the multimeric structure of Nme5-likeCc. However, the multimeric formation of the Nme enzyme that exceeds conjugation of more than six monomers has not yet been described. Adenylate kinase enzymes can form both dimers and higher supramolecular structures [74,75]. The crystal structure of the adenylate kinase from maize revealed that the enzyme can form rods that are linear assemblies of hexamers. It was proposed that the supramolecular assembly into rods occur in vivo overnight in mesophyll chloroplasts. Presumably rods represent a natural inactive storage form that assembles at night when maize stops CO_2_ assimilation, i.e., most of the AMP production via C_4_ cycle [75]. This supramolecular assembly might have originated very early in plant evolution and might also be connected to a C_4_-like carbon-fixation pathway that might be present in red algae. Most of the essential genes related to the C_4_-pathway are present in red algae and special C_4_-like carbon-fixation pathway might play an important role in the conchocelis stage of Rhodophyta [76,77]. It is yet to be determined if the supramolecular assembly of the Nme5-likeCc is characteristic for particular developmental stage during the life cycle of *C. crispus*.

Our results show that besides the role in the maintenance of the cellular NTP pool, Nme5-likeCc probably has an impact on cell proliferation. It was suggested that NF-κB is a key executor of human Nme5 in regulating apoptosis and the cell cycle. Human Nme5 was capable of binding NF-κB p65 and affecting its expression level, suggesting that Nme5 probably acted upstream of NF-κB p65 pathway. However, the overexpression of Nme5-likeCc did not induce apoptosis (data not shown) in HEK293T cells, which may suggest that the mechanism underlying this effect was established later in metazoan evolution. These results support the idea that the algal protein has a different cellular function in comparison to its human homolog. Therefore, we conclude that the structure of the ancestral *Nme5* gene has changed during metazoan evolution, possibly in correlation with the function of the protein.

## 4. Materials and Methods

### 4.1. Distribution of the Nme Protein Family Across Eukaryotic Supergroups and Phylogenetic Analyses

Eukaryotic Nme proteins from model organisms with fully sequenced genomes were obtained from the NCBI non-redundant (NR) database (http://blast.ncbi.nlm.nih.gov/Blast.cgi). Additionally, BLASTP on the Ensembl database (http://www.ensembl.org/index.html) were used. Genomes were also searched at the http://www.broadinstitute.org/annotation/genome/multicellularity_project/GenomesIndex.html. Domain architectures of retrieved sequences were obtained from the databases Pfam (http://www.sanger.ac.uk/Software/Pfam) and SMART (http://smart.embl.de/) and examined through the NCBI conserved domain search website (http://www.ncbi.nlm.nih.gov/Structure/cdd/cdd.shtml). The 3D structures of the ADK, Dpy-30, and NDK domains were modeled by homology-modeling using SWISS-MODEL (http://swissmodel.expasy.org//SWISS-MODEL.html) and molecular presentations were generated using the PyMOL program (http://www.pymol.org).

The collected amino acid sequences were aligned with MUSCLE (Multiple Sequence Comparison by Log- Expectation) multiple alignment tool, using default settings [78]. The multiple alignment was subjected to maximum-likelihood (ML) analysis using MEGA6 (Molecular Evolutionary Genetics Analysis) [79], while the Bayesian MCMC analysis was conducted in MrBayes 3.1.2 [80]. The model for the ML analysis was selected with ProtTest 2.4 and the Akaike information criterion (AIC) [81], which indicated a Le_Gascuel model (I+G) [82]. Bootstrap tests were performed with 1000 replicates.

### 4.2. Isolation of Genomic DNA from Chondrus Crispus and Nme5-Like Gene Cloning

The genomic DNA from the red alga, *C. crispus* (Gigartinales, Rhodophyta) was extracted using DNeasy Plant Mini Kit (Qiagen, Hilden, Germany), according to the manufacturer’s protocols, with the following modifications: 100 mg of frozen tissue was ground in liquid nitrogen and resuspended in 800 µL of lysis buffer. Samples were vigorously vortexed for several minutes and incubated for 30 min at 65 °C with shaking. A centrifugation step was added to remove potential clumps. Approximately 5 µg of DNA was obtained from 100 mg of tissue powder.

For the overproduction of recombinant Nme5-likeCc protein 5′-CTGCACATATGGTCGGCCTGCAAATCGAG-3′ and 5′-ATATGGATCCTTTTAAAAAAGGCCGATCAC-3′ set of primers were used to amplify *Nme5-likeCc*. PCR product and pET-15b expression vector were ligated after NdeI/BamHI restriction.

For the localization assay, *Nme5-likeCc* was cloned in fusion with EGFP into pEGFP-C1 (XhoI/BamHI restriction sites) using the following set of primers: 5′- CCCACTCGAGCTATGGTCGGCCTGCAAATCG-3′ and 5′-ATATGGATCCTTTTAAAAAAGGCCGATCAC-3′.

For cell proliferation, apoptosis, and soft agar colonization, assay mammalian expression vector pcDNA3 was used to clone *Nme5-likeCc* into BamHI/EcoRI restriction sites using the following set of primers: 5′-CTAGGGATCCACGAGATGGACTACAAGGACGACGACGATAAGATGGTCGGCCTGCAAATC-3′ and 5′-GAATATGAATTCTTTTAAAAAAGGCCGATCAC-3′.

All PCR reactions were performed using Q5 High-Fidelity DNA Polymerase (NEB). Plasmid constructs were verified by sequencing (ABI PRISM 3100-Avant Genetic Analyzer, Applied Biosystems, Foster City, CA, USA) using ABI PRISM BigDye Terminator v.3.1 Ready Reaction Cycle Sequencing Kit (Applied Biosystems, Foster City, CA, USA).

### 4.3. Expression and Purification of the Recombinant Nme5-LikeCc Protein and Western Analyses

The soluble recombinant His-Nme5-likeCc protein, visible after SDS-PAGE, was obtained under the following growth conditions: *Escherichia coli* strain BL21(DE3) harboring the plasmid was grown to OD_600_ 1.2 and protein expression was induced with 0.05 mM IPTG for 1 h at 30 °C. Bacteria were collected by centrifugation, resuspended in lysis buffer (50 mM Tris HCl, 300 mM NaCl, 10 mM imidazole, and 1 mg/mL lysozyme), incubated 30 min on ice and sonicated 8 × 30 sec (50% of full power). After 25 min of centrifugation (12,000 rpm) at 4 °C, the supernatant was applied onto cobalt-charged agarose column (TALON). Histidine tagged proteins was eluted with 300 mM imidazole. Collected eluates were separated by SDS-PAGE (Bio-Rad, Hercules, CA, USA) and electrotransfered onto PVDF Hybond-P membrane (Amersham Biosciences, Buckinghamshire, United Kingdom). For the detection of His-Nme5-likeCc, the membranes were incubated with anti-His antibody (Amersham Biosciences, Buckinghamshire, United Kingdom). Protein bands were visualized using chemiluminescence detection (Amersham ECL Plus, GE Healthcare Chicago, IL, USA). Nme5-likeCc protein was concentrated and desalted using Amicon Ultra 10K device (Millipore, Burlington, MA, USA). The same Ultracel was used for buffer exchange (Nme buffer, [53]).

### 4.4. Oligomerization of Protein and Gel Filtration Chromatography

The oligomerization of the recombinant Nme5-likeCc protein was performed in PBS buffer as follows: 300 ng of protein were pre-incubated in PBS at room temperature and various concentrations of glutaraldehyde were added to initiate the cross-linking. Following 15 min incubation, the reaction was quenched with 0.2 M Tris-HCl, pH 7.5, for 15 min at room temperature. The reaction product was subjected to 12% SDS-PAGE, transferred onto PVDF Hybond-P membrane (Amersham Biosciences, Buckinghamshire, United Kingdom) and visualized as described before. Human Nme2 protein was used as a control.

Gel filtration chromatography using the fast protein liquid chromatography (FPLC) system was employed for the determination of the protein MW. Recombinant Nme5-likeCc was loaded onto HiLoad 26/600 Superdex 200 prep grade column and eluted with Nme buffer [53] at a flow rate of 1 mL/min. The standard protein markers for gel filtration chromatography were as follows: ferritin (440 kDa), β-amylase (200 kD) and alcohol dehydrogenase (150 kD).

### 4.5. NDP Kinase Assay

NDPK activity was measured using a coupled pyruvate kinase—Lactate dehydrogenase (PK-LDH) assay [83,84] in the presence of various phosphate donors (ATP, dATP, GTP, and dGTP) and dTDP as phosphate acceptors. Reaction mixtures comprised 50 mM Tris-HCl pH 7.4, 75 mM KCl, 5 mM MgCl_2_, and 1 mM phosphoenolpyruvate, 0.1 mg/mL NADH, 1 mM (d)NTP, 0.2 mM dTDP, 2 U/mL of PK (from rabbit muscle, SIGMA, St. Louis, MO, USA), 2.5 U/mL of LDH (from rabbit muscle, SIGMA, St. Louis, MO, USA) and 1 mg/mL BSA. Reactions were initiated by the addition of 50 ng of Nme5-likeCc enzyme and the activity was monitored in an Ultrospec 2100 pro (Amersham Pharmacia Biotech, Amersham, Buckinghamshire, United Kingdom) by measuring the decrease in absorbance at 340 nm. All reactions were performed in triplicate. Results are presented as an average of three measurements ± standard deviations.

### 4.6. DNA-Binding Assay

The affinity of recombinant Nme5-likeCc to various types of DNA was tested in vitro. Reactions contained 10 ng of single-stranded circular DNA from bacteriophage φX174 (NEB) and 10 ng of double-stranded circular DNA (bacteriophage φX174 RF I (NEB)). The amount of purified protein is indicated (Figure 2). BSA was used as a control. The reaction mixture was incubated in 20 μL of buffer containing 100 mM KCl, 10 mM MgCl_2_, 50 mM Tris HCl pH 7.5, 0.2 mM DTT, 10 µg/mL BSA. After 45 min of incubation at 37 °C, products were subjected to gel electrophoresis in 1% agarose and stained with SYBR gold (Invitrogen). Thirty nanograms of double-stranded 57-bp nuclease hypersensitive element of the *c-myc* promoter (5′-GATCCCCAGTCTCCTCCCCACCTTCCCCACCCTCCCCACCCTCCCCATAAGCGAATT-3′), as well as 30 ng of single-stranded telomeric DNA (TTAGGG)_6_ were incubated with recombinant protein in the same reaction buffer. After 30 min of incubation at 37 °C, products were subjected to gel electrophoresis in 4% agarose and stained with SYBR gold (Invitrogen, Carlsbad, CA, USA).

### 4.7. Transient Transfections and Laser Scanning Confocal Microscopy

HeLa and HEK293T cells were used for transfection by Lipofectamine 3000 reagent (Invitrogen), according to the manufacturer’s instructions. Twenty-four hours prior to transfection, 5 × 10^4^ cells were seeded onto 24-well culture slides containing DMEM supplemented with 10% FBS and grown until 80% confluent. Cells were transfected with 500 ng of plasmid DNA. Forty-eight hours post transfection, the cells were washed with PBS pH 7.5, fixed in 4% formaldehyde, and mounted in SlowFade Diamond Antifade Mountant, with or without DAPI (Molecular Probes, Eugene, OR, USA).

Fluorescent images were obtained using a Leica SP8 X FLIM laser scanning confocal microscope (Leica Microsystems, Wetzlar, Germany) equipped with HC PL APO CS2 63×/1.40 OIL objective. GFP was excited by 488 nm, CFP using 433 nm, and DAPI using 405 nm laser lines.

### 4.8. Soft Agar Colonization Assay

HEK293T cells were transfected as described above with FLAG-Nme5-likeCc or empty pcDNA3 expression vector in triplicate. Forty-eight hours post-transfection cells were suspended in 1 mL 10% FBS DMEM, containing 0.36% agar and layered on a 0.75% agar in a 6-well plate at a concentration of 5 × 10^3^ cells/well. Cells were incubated for 16 days at 37 °C and 5% CO_2_. Colonies were stained with 0.005% Crystal Violet and counted.

### 4.9. Cell Proliferation Assay

Cells were transfected as described above, trypsinized 24 h post-transfection, and 10^3^ viable cells suspended in 90 µL 10% FBS DMEM were added to each well of 96-well plates. Cell proliferation was measured using CellTiter-Glo Luminescent Cell Viability Assay (Promega, Madison, Wisconsin, United States) according to the manufacturer’s instructions with luminometer Infinite 200 (TECAN, Männedorf, Switzerland). The concentration of ATP was measured in triplicate, and error bars represent the 95% confidence interval. The expression of FLAG-Nme5-likeCc was checked by Western blot analyses with anti-FLAG M2 antibody (1:5000) (Sigma-Aldrich, St. Louis, MO, USA).

### 4.10. Detection of Apoptosis

Cells were transfected as described above, trypsinized 24 h post-transfection, and 5 × 10^3^ viable cells suspended in 100 µL 10% FBS DMEM were seeded in 96-well culture plates. Caspase-3/7 activity was measured using the Caspase-Glo 3/7 Assay Kit (Promega, Madison, Wisconsin, United States) according to the manufacturer’s instructions with luminometer Infinite 200 (TECAN, Männedorf, Switzerland).

### 4.11. Statistical Analyses

Statistical analysis was performed using Student’s *t*-test. Probabilities of less than 0.05 were considered statistically significant.

## Figures and Tables

**Table 1:** Schematic architecture of Nme proteins present in eukaryotic representatives. Numbers indicate amino acids. Proteins are represented in a scale 1:10 (1 mm = 10 amino acids). Protein domains have been indicated with colored boxes and each protein has been searched against SMART (Simple Modular Architecture Research Tool)/Pfam databases. Abbreviations of domain names are retrieved from SMART/Pfam databases and indicated in the figure. Shortened names include: NDK, nucleoside diphosphate kinase; ADK, adenylate kinase; Dpy-30, domain found in the Dpy-30 proteins; DM10, uncharacterized domain; IQ, calmodulin-binding motif; TM, transmembrane region; Domain which is not retrieved from SMART/Pfam database: MLS, mitochondrial localization signal according to [52].

	Eukaryotic Supergroup	Organism	Nme Schematic Domain Presentation	
			Group I	Group II
Unikonts	Opistokonts	*Homo sapiens*	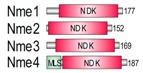	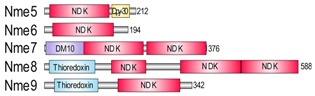
		*Spizellomyces punctatus*	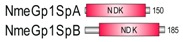	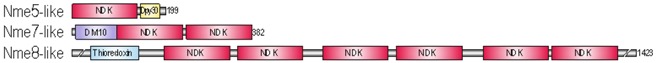
	Apusozoa	*Thecamonas trahens*	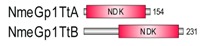	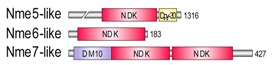
	Amoebozoa	*Dictyostelium purpureum*	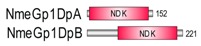	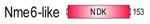
Bikonts	Rhizaria	*Bigelowiella natans*	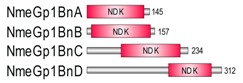	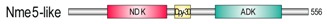
	Chromalveolata	*Phytophthora sojae*	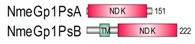	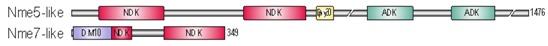
	Plants	*Volvox carteri*	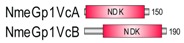	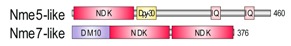
		*Chondrus crispus*	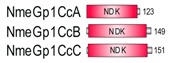	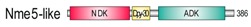
	Excavata	*Naegleria gruberi*	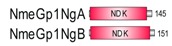	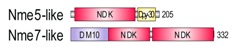
Eukaryotic ancestor			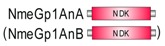	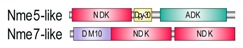

## Supplementary Materials

The following are available online at https://www.mdpi.com/1660-3397/18/1/13/s1, Figure S1: Crystal structure of human nucleoside diphosphate kinase A S120G complexed with ADP (PDBID: 2HVE1) (orange, A), overlaid with human Nme5 (green, B) or Nme5-likeCc (white, C). Arrows indicate the ADP-binding cavity. Note disturbances produced by 3 aa insertion in Kpn loop in human Nme5 indicated by yellow arrows, Table S1: The percentage of identical amino acids and overall sequence similarity between NDPK domains.
