# Supplementary material for: Characterization of Nme5-Like Gene/Protein from the Red Alga Chondrus Crispus
Source: Mar Drugs. 2019 Dec 21;18(1):13. doi: 10.3390/md18010013 (PMC7024210; doi:10.3390/md18010013)
Supplement: Supplementary file 1 [file marinedrugs-18-00013-s001.pdf]

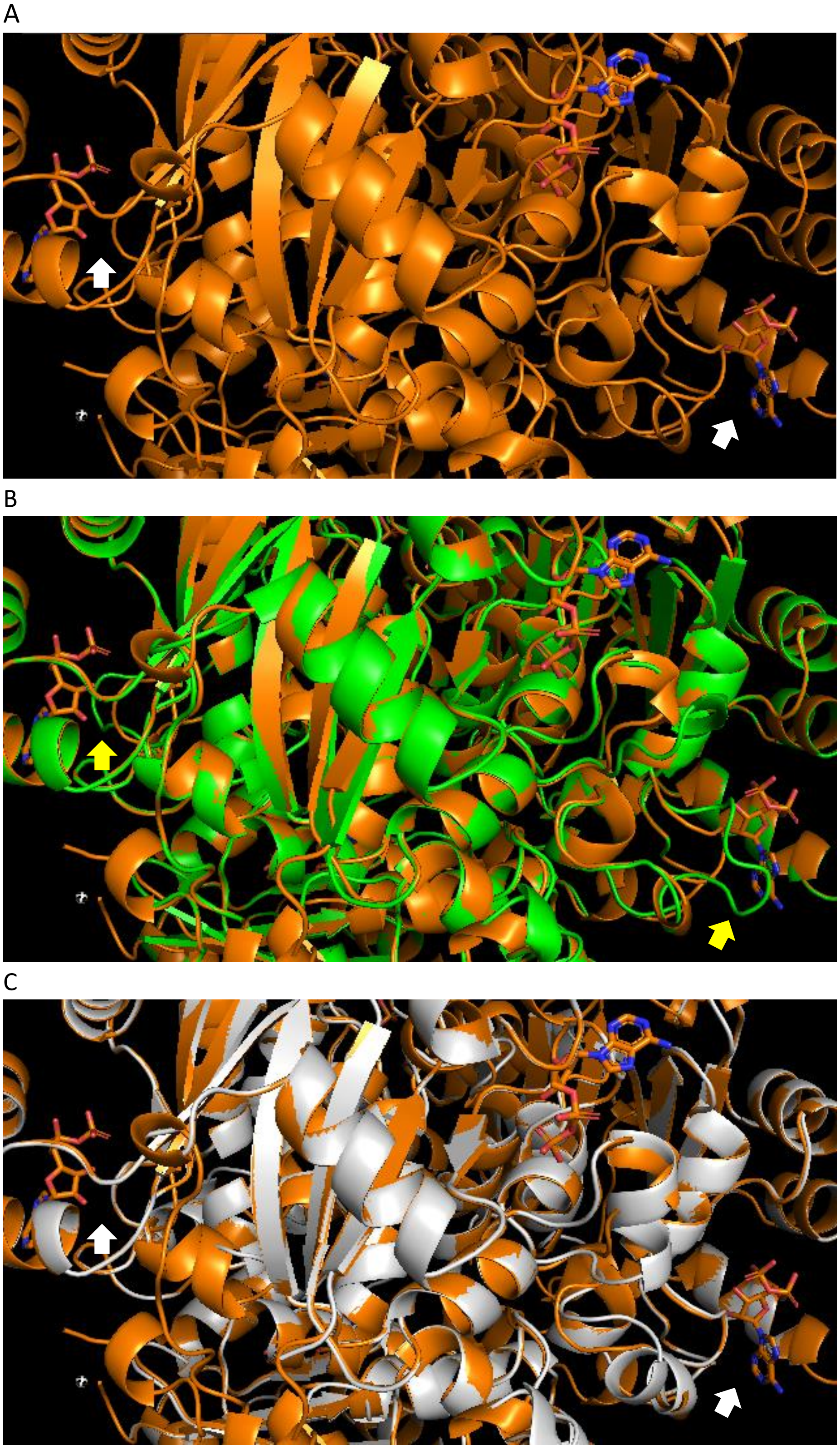

Figure S1. Crystal structure of human nucleoside diphosphate kinase A S120G complexed with ADP (PDBID: 2HVE1) (orange, A), overlaid with human Nme5 (green, B) or Nme5-likeCc (white, C). Arrows indicate the ADP-binding cavity. Note disturbances produced by 3 aa insertion in Kpn loop in human Nme5 indicated by yellow arrows.

Table S1. The percentage of identical amino acids and overall sequence similarity between NDPK domains

	Nme1Hs	Nme2Hs	Nme3Hs	Nme4Hs
Nme5Hs	29%/46%	31%/49%	29%/46%	29%/48%
Nme5Cc	31%/48%	30%/51%	31%/51%	31%/48%
